# Development of a tool box for mesenchymal stem cell secretome analysis

**DOI:** 10.1186/1753-6561-9-S9-P66

**Published:** 2015-12-14

**Authors:** Cornelia Kasper, Anna Koch, Verena Charwat, Martina Marchetti-Deschmann

**Affiliations:** 1 University of Natural Resurces and Life Science, Vienna, Austria; 2 Vienna University of Techchnology, Vienna, Austria

## Background

The development of optimized cultivation strategies for mesenchymal stem cells (MSC) is one major field in the area of tissue engineering and cell based therapies. The physiological tissue niche of stem cells is characterized by a complex set of structural, physical and chemical cues, which create a complex three-dimensional dynamic microenvironment. Mimicking these conditions in vitro is a promising strategy for expansion and differentiation of MSC. This also includes the application of physiological oxygen concentrations (e.g. 3-8 % for most tissues).Multiple cell biological effects of varying oxygen supply have been reported [1]. However, a lot of current research work is dedicated to the analysis of selected biomarkers and distinct signaling pathways, which carries the risk of overlooking unexpected biological effects. A more systemic approach involves the investigation of the entirety of proteins secreted by the cells - the secretome. However, secretome analysis is very challenging for most current cell cultures, because serum or other undefined protein-rich medium supplements are commonly used and create a strong background of high-abundance proteins in the samples. In order to exploit the potential of secretome analysis for MSC cultures a methodological toolbox was developed that provides access to the rather low abundant secretome in serum containing media.

## Experimental approach

MSC derived from adipose tissue were cultivated under hypoxic (5% O2 ) and normoxic (21% O2) conditions in αMEM medium supplemented with 10% human serum. Cell culture supernatants were collected after 4 days incubation on a confluent cell layer, corresponding to approximately 700 000 cells/ml. During sample preparation proteins were precipitated with trichloroaceticacid / acetone (v:v1:8) dissolved in IPG-buffer (7M Urea, 2MThiourea, 2% CHAPS) and protein concentration was determined by Bradford assay. Reproducibility of sample preparation was tested by repeated preparation of 2D gels. Furthermore, we compared two depletion methods (top 12 depletion columns, Pierce and combinatorial hexapeptide library, BioRad) for reduction of high abundant proteins to find a well reproducible strategy that allows analysis of the lower abundant secreted proteins. The proteomics approach was applied to characterize the MSC secretome after removal of high abundance proteins by affinity enrichment and depletion.

## Results

**Figure 1:**
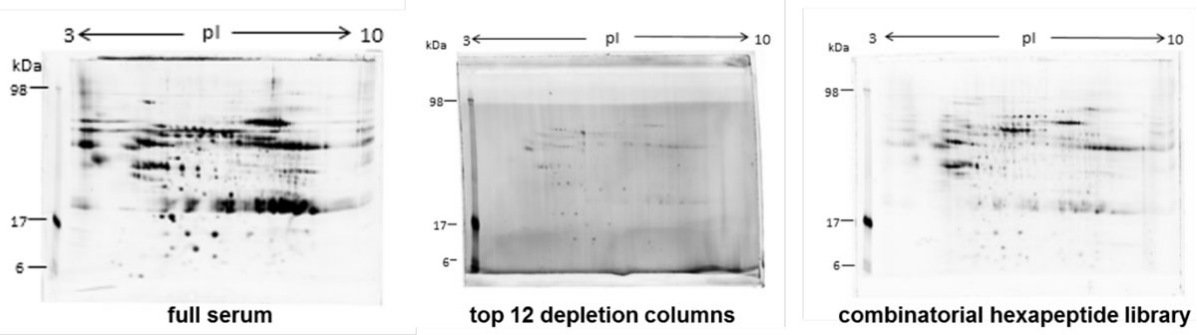
**2DSDS-PAGE comparison of the two depletion methods**.

The first step was the evaluation of the method for protein precipitation and 2D gel electrophoreses, which revealed highly reproducible sample preparation. However, the visualization and detection of low-abundance proteins remained difficult. Therefore, high-abundance serum proteins had to be significantly removed without losing valuable secreted proteins. An effective depletion of serum proteins and thus reduction of high-abundance proteins was observed resulting in a reduced number of protein bands in 1D and a lower number of spots in 2D PAGE was achieved after utilizing Top12 depletion spin columns. The possibility of co-depletion of low abundant proteins was considered to be very likely. Therefore the combinatorial peptide library was also tested, but showed a lower efficiency for serum protein removal (Figure 1).

## Conclusions

Serum containing cell culture supernatants could be reproducibly plotted on 2D gels but removal of high abundant proteins was required for the intended secretome analysis. Both tested methods efficiently reduced high abundance serum proteins giving access to the interesting secretome of MSC. Future work will focus on DIGE analysis to point out proteome differences between the normoxic and hypoxic secretome of MSC and differently expressed proteins will be identified by mass spectrometry.
